# Ovarian tissue grafting: Lessons learnt from our experience with 55 grafts

**DOI:** 10.1002/rmb2.12380

**Published:** 2021-05-12

**Authors:** Genia Rozen, Stephanie Sii, Franca Agresta, Debra Gook, Alex Polyakov, Catharyn Stern

**Affiliations:** ^1^ Reproductive Services Unit Royal Women's Hospital Parkville, Melbourne Vic. Australia

**Keywords:** assisted reproductive technology, cryopreservation of germ cells, endocrinology, fertility of female, oncology

## Abstract

**Purpose:**

Uncertainties remain regarding the clinical efficacy of ovarian tissue cryopreservation and grafting. We report a retrospective analysis of reproductive outcomes and lessons learnt following 55 ovarian tissue transplant procedures at our center from 2006 to 2019.

**Methods:**

We analyzed variables related to graft success such as tissue volume, follicular density, total follicular volume, and age on the duration of graft function.

**Results:**

Follicular density and total follicular volume correlate positively with duration of graft function. All clinical pregnancies in our cohort occurred in women who were aged 35 or less at the time of ovarian tissue cryopreservation.

**Conclusion:**

Graft success, as determined by eventual pregnancy and the longevity of graft function, may be impacted by factors including age at cryopreservation, follicular density, and total follicular volume.

## 1 INTRODUCTION

A major side effect of cancer treatment in young women, such as chemotherapy and radiation therapy, is the loss of ovarian function and fertility, which has a significant impact on quality of life.^1^ Given the relatively high incidence of cancer in reproductive age women and improvements in survival, an increasing number of women are presenting for discussion of ways to preserve their fertility prior to undergoing gonadotoxic treatment.^2^ A number of strategies have been developed in recent years, including medical therapies to suppress ovarian function, improvements in cryopreservation of oocytes or embryos, and ovarian tissue cryopreservation (OTC).^3^

Ovarian tissue cryopreservation can be performed at any time without delaying the cancer treatment and is the only available option to preserve fertility in children and premenarchal adolescent girls.^4^ Harvesting part or a whole ovary allows the cryopreservation of potentially thousands of oocytes in their immature form, whereas oocyte freezing will result in storage of only a finite number of mature oocytes with a limited number of potential opportunities.^5^ The procedure may not only benefit those with oncological diseases, but also those with benign diseases that require conditioning regimens for bone marrow transplant (BMT) such as aplastic anemia or thalassemia or those with autoimmune conditions and genetic disorders associated with the development of premature ovarian insufficiency (POI).^6^, ^7^ Since the first study that described OTC and grafting in 2000^8^ and first live birth reported in 2004,^9^ over 140 babies have been born worldwide.^3^, ^10^

However, uncertainties remain regarding its clinical efficacy, optimal grafting site, tissue volumes, and factors which may predict success.^5^ As thousands of women have had ovarian tissue frozen, increasing numbers are returning for ovarian tissue grafting, making exploration of these issues critical.^11^ We report a retrospective analysis of reproductive outcomes following 55 ovarian tissue transplant procedures at our center from 2006 to 2019, and analyze variables related to graft success such as tissue volumes and follicular density.

## 2 MATERIALS AND METHODS

Initially, tissue freezing was approved under Human Research and Ethics provided by Royal Women's Hospital (RWH). Following extensive validation of procedures in xenografted models,^12^ Clinical Review Board of RWH gave permission for clinical transplantation pilot study. Informed consent was obtained from each patient before cryopreservation and transplantation, with counselling about their fertility preservation options, procedures involved and surgical risks. In addition, oocyte or embryo freezing was performed in some patients with appropriate consent obtained. Patients with oncological indications for fertility preservation were counselled regarding the possibility of tumor cell transmission following future grafting procedure.

A total of 629 patients had cryopreservation at our center between 1994 and 2019, with patients requesting grafting representing 4.6% (29/629) of these. A total of 55 grafts were performed in 40 patients, of whom 29 had their ovarian tissue harvested and cryopreserved at our center and 11 in other centers. The age of the patients at the time of tissue harvest and freezing was between 17.8 and 40.7 years (median age 26 years).

The oncological (81%) and medical (19%) indications for ovarian tissue freezing are shown in Table 1. Non‐oncological indications included imminent POI, severe endometriosis, benign ovarian cysts, and autoimmune conditions such as Wegener's granulomatosis and systemic lupus erythematosis, which may have the potential to compromise future fertility. Oncological indications included Hodgkin's and non‐Hodgkin's lymphoma, granulosa cell ovarian tumor, breast, uterine and cervical cancer, Ewing's sarcoma, and soft tissue sarcoma. Ovarian tissue harvest was performed laparoscopically, with the exception of those requiring laparotomy for treatment of their medical condition. The tissue was harvested prior to gonadotoxic treatment in all but 10 patients (Table 1).

**TABLE 1:** Patient characteristics and grafting details

Patient No.	Age at cryopreservation	Prior chemo‐therapy	Disease	Uterus affected by treatment	Requires gestational carrier	Freezing at our institution	Year of cryopreservation	Duration of cryopreservation	Year of transplantation	Graft sites^a^
1	17.8	Yes	Non‐Hodgkin’s lymphoma	TBI		Y	1996	10.5/12.1	2006/2008	A+P+O/A+P+O
2	19.0		Sarcoma soft tissue			Y	2004	14.2	2018	A+P
3	19.5		Benign ovarian cysts			N	2000	9.1/11.6	2011/2012	A/A+P
4	21.6		Cervical cancer	PRT	Yes	Y	2009	3.6/5.6	2012/2014	A+P/A+P
5	22.0		Hodgkin’s lymphoma			N	2009	6.7	2015	A+P
6	22.0	Yes	Ewing’s sarcoma			Y	2014	3.1	2017	A+P
7	22.2		Endometriosis		Yes	N	1997	13.3/16.6/18.2	2010/2013/2015	A+P/A+P/A+P
8	23.3	Yes	Hodgkin’s lymphoma			Y	2013	6.7	2020	A+P+O
9	23.5		Non‐Hodgkin’s lymphoma	TBI		Y	2009	3.9	2013	A+O
10	24.7		Granulosa cell tumor			Y	2003	7.3/9.4	2010/2012	A+P/A+P
11	25.0		Wegener’s granulomatosis			Y	1997	16.7	2014	P
12	25.4		Hodgkin’s lymphoma			N	2011	8.7	2019	A+P+O
13	25.5		Autoimmune polyarteritis			Y	2005	13.1	2019	A +P+O
14	26.1		Pelvic sarcoma	PRT	Yes	Y	2005	7.8	2013	A
15	26.1	Yes	Hodgkin’s lymphoma			N	2000	12.5	2012	P
16	26.9		Hodgkin’s lymphoma			Y	2009	10.6	2019	A+P+O
17	28.1	Yes	SLE, lupus nephritis			Y	1996	11.8	2008	A+O
18	29.0	Yes	Anti‐NMDA encephalitis			N	2015	2.6	2018	A+P
19	30.0		Cervical cancer		Yes	N	2017	1.9	2018	A
20	30.7		Wegener’s granulomatosis			Y	1999	8.3/10.0	2007/2008	A+P+O/P+O
21	31.0	Yes	Hodgkin’s lymphoma			Y	2011	4.7	2016	A+P
22	31.0		Neuroendocrine tumor of cervix		Yes	Y	2013	2.2/3.6	2015/2017	A/A
23	31.4		Cervical cancer	PRT	Yes	Y	2011	2.4/3.8	2013/2014	A/A
24	31.7	Yes	Hodgkin’s lymphoma			Y	2010	4.1/6.3	2014/2016	A+P/A+P
25	31.9	Yes	Non‐Hodgkin’s lymphoma	TBI		Y	2005	3.7/6.4	2008/2011	A+P+O/P+O
26	32.6		Cervical cancer	PRT	Yes	Y	2011	1.8	2012	P+O
27	32.9		Ewing’s sarcoma			Y	2019	4.3	2019	A+P
28	33.0		Cervical adenocarcinoma		Yes	Y	2010	5.8	2016	A+P
29	33.0		Cervical cancer		Yes	N	2007	10.4	2018	A+P
30	33.5		Cervical cancer			Y	2016	2.1/3.6	2018/2019	A/A
31	34.1		Hodgkin’s lymphoma			Y	2015	3.9	2019	A+P+O
32	34.2		Endometrial cancer		Yes	N	2010	3.2	2013	A+P
33	35.7		Endometriosis			Y	1997	11.5	2008	A+P+O
34	36.0	Yes	Non‐Hodgkin’s lymphoma			Y	2014	3.3/4.4	2017/2018	A+P/P
35	36.0		Endometrial sarcoma		Yes	Y	2014	3.0	2017	A+P
36	37.1		Breast cancer			Y	2005	7.6/10.4	2013/2015	P+O
37	37.4		Premature ovarian insufficiency^b^			N	2001	7.1	2009	A+P+O
38	38.4		Breast			N	2017	2.0	2019	A+P+O
39	39.0		Cervical cancer			Y	2016	1.5/2.7	2017/2019	A+P
40	40.7		Desmoid tumor			Y	2016	3.1	2019	A+P

Abbreviations: N, No; PRT, pelvic radiotherapy; TB, total body radiation; Y, Yes.

^a^ A=abdominal wall, P=pelvic side wall, O=ovary.

^b^ Patient 19 had tissue frozen interstate and the indication appears uncertain and documented as premature ovarian insufficiency.

### 2.1 Statistical analysis

Descriptive statistics are used throughout to describe various outcomes. Statistical analysis was restricted to determining correlation coefficients and was performed by calculating Pearson's correlation coefficient and producing scatter plots with lines of best fit to visually demonstrate the strength of association. STATA version 16.1 (StataCorp) was used.

### 2.2 Collection and cryopreservation of ovarian tissue

In three patients, unilateral oophorectomy was performed due to high risk of POI from impending BMT and radiotherapy treatment. Partial oophorectomy was performed in the remainder, avoiding the use of electrocoagulation. No surgical complications such as infection, bleeding, or organ damage were observed in this group of patients. Histology and immunohistochemistry, where appropriate, were performed on a sample of retrieved tissue to exclude malignant infiltration and to estimate follicle density. Follicular density was estimated as the average number primordial follicles counted in at least two pieces of cortex per millimeter of ovarian cortex. There was no evidence of malignancy in any samples of the tested tissue. The ovarian tissue was trimmed to 1 mm depth and subsequently sliced (approximately 1 mm wide by 3‐4 mm long) to aid cryoprotectant (propanediol) penetration and cryopreserved according to our previously published protocols.^12^, ^13^, ^14^ Larger pieces of cortex (5 × 5 × 1 mm) were also frozen in some cases. Vials containing multiple slices were stored in nitrogen vapor, and for these patients, the duration of storage was between 1.5 and 16.7 years (Table 1). For tissue cryopreserved at other centers (Table 2), less detailed information regarding the volume of tissue or size of pieces or cryopreservation procedure was available. Slow freezing was used for all cases regardless of center where freezing was performed; however, no specific details were given apart from cryoprotectants used at the other center: propanediol (patient 3, 5, 15, 18, 19, 29), dimethyl sulfoxide (patient 7,12, 32,37), and ethylene glycol (patient 38).

**TABLE 2:** Transplantation details and outcomes for Group 2 (tissue frozen at other centers)

Patient No.	Volume tissue grafted (mm^3^)	Tissue density (follicle/mm^2^)	Time to resumption (mo)	Duration of function (mo)	Treatment details
41	125/250	0.1	5/0	13/—	Lost to follow‐up
42	450/1800/165	1.2	4/4/1	36/16/17	In treatment
43	—	—	0	—	Ceased treatment
44	—	—	3	—	Lost to follow‐up
45	350	—	5	6	Ceased treatment
46	450	4.2	1	24	In treatment
47	150	0.6	4	6+	In treatment
48	40	0	2	6	In treatment
49	1350	2.7	8	19+	In treatment
50	690	0	0	—	Ceased treatment
51	270	0.5	2	6+	In treatment

### 2.3 Transplantation of thawed ovarian tissue

Grafting of stored ovarian tissue was considered for patients who presented with POI requesting fertility assistance. The age of the patients at the time of first grafting ranged between 25.1 and 47.2 years (median age 36). Two patients were particularly interested in restoration of endocrine function in addition to or instead of a specific desire for fertility. Menopausal status was confirmed by cessation of menstruation and persistently elevated FSH over 40 IU/L in all the patients. AMH levels were below 0.1 pmol/L in all patients. The patients' medical fitness was considered and in all cases suitability to proceed with fertility and pregnancy was confirmed by the treating medical specialist.

In situations where there was concern about uterine functionality for pregnancy following radiation, pelvic ultrasound, MRI and the adequacy of endometrial response to artificial hormonal stimulation were utilized to assess uterine functionality for pregnancy.

Prior to grafting, a small amount of frozen cortex and a was thawed and subjected to detailed histopathology and immunohistochemistry where appropriate. No malignancies were detected in any of the samples examined. The ovarian tissue was rapidly thawed and rehydrated as previously described.^15^

### 2.4 Graft sites

In patients with tissue frozen at our center, ovarian slices were threaded onto a 6.0 vicryl suture horizontally to maximize area of contact with peritoneum to facilitate neovascularization (generally 10‐15 slices per suture) with total tissue volumes ranging 60‐575 mm^3^ (Table 3). The total number of slices available determined the amount of tissue put back in the first procedure (generally half of total frozen), to leave some tissues available for future use. Ovarian tissue was laparoscopically grafted into multiple sites including the remaining ovary, pelvic side wall, and abdominal wall. Lateral ports are paced 5‐8 cm supero‐medially to the anterior superior iliac spines bilaterally. The ports are then gently withdrawn from the abdominal cavity with the internal opening just superior to the peritoneal layer. A peritoneal pocket or tissue tunnel is then made with a non‐toothed laparoscopic forceps. The tissue graft is pushed into this created extraperitoneal tunnel, at which point the port is fully withdrawn and closed with suture where necessary. Grafting to the atrophic ovary was used for some and tissue placed within a 1‐2 cm antimesenteric pocket created and similarly closed with suture. The grafting sites used were largely determined at the time of operation, depending on pelvic anatomy (such as the presence of adhesions) and past treatment, such as radiotherapy. Volume of tissue grafted could not be estimated for patients 43 and 44 (Table 2) due to tissue having been minced into small fragments these were introduced to pockets using a pipelle.

**TABLE 3:** Transplantation details and outcomes for Group 1 (tissue frozen at our institution)

Patient No.	Volume tissue grafted (mm^3^)	Follicle density (follicle/mm^2^)	Time to resumption (mo)	Duration of function (mo)	Treatment details
1	84/168	14.3	7/3	18/65	Ceased treatment
2	531	1.8	5	9+	In treatment
4	160/155	5.7	7/1	10/4+	In treatment
6	545	1.1	1	14+	In treatment
8	405	6.7	3.0	2+	In treatment
9	255	4.5	4	12+	Ongoing pregnancy
10	295/150	7.6	4	39/—	Ceased treatment
11	60	1.9	7	2	Ceased treatment
12	430/675	0.3	5	9+/—	In treatment
13	440	2.0	4	5	In treatment
14	575	1.3	5	11+	Ceased treatment
16	240	0.7	4.3	7+	In treatment
17	270	2.2	5	10	In treatment
20	125/250	0.1	5/—	13/—	Ceased treatment
21	243	1.6	5	9	In treatment
22	404/295	5.4	3/1	12/20+	In treatment
23	170/280	0.6	6/5	2/6+	Ceased treatment
24	250/295	5.5	5/3	12/29	In treatment
25	205/235	1.4	5/—	36/—	Ceased treatment
26	170	7.5	4	9	Passed away
27	450	2.2	5.4	6+	In treatment
28	235	0.6	4	16	Loss to follow‐up
30	410/350	0.2	2/4	6/—	In treatment
31	240	3.9	5.3	6+	In treatment
33	387	0.7	2	14	Ceased treatment
34	160/170	0.2	9/—	1/—	In treatment
35	270	6.4	4	22+	In treatment
36	260/305	7.5	3/1	29/—	Loss to follow up
40	315	0.2	6.3	1	In treatment

### 2.5 Monitoring

The management plan for patients following graft surgery, involved ultrasound and endocrine assessment including follicle stimulating hormone (FSH), luteinizing hormone (LH), progesterone (P4) and estradiol (E2) at 4 weeks and monthly thereafter. When endocrine activity resumed, as indicated by a drop in FSH (<20 U/ml) and rise in estradiol levels (>200 pg/L), ovarian function was monitored with ultrasound and endocrine assessment tracked for one to three cycles, after which time an IVF cycle was commenced if appropriate for those patients requesting fertility.

## 3 RESULTS

### 3.1 Restoration of ovarian activity

Of the 55 grafts (Table 1), 40 grafts belonged to the 29 patients who underwent cryopreservation at our center (Group 1; Table 3) and 14 grafts were from the 11 patients who had tissue cryopreserved in other units (Group 2; Table 2). Ovarian activity as demonstrated by a rise in serum E2 and fall in FSH, occurred in 36/40 patients (90%) after a mean of 4.5 months (range 1‐9 months). In Group 1, activity was observed in 29/29 patients (100%; Table 3). For those patients in Group 2, activity resumed in 9/11 patients (82%; Table 2).

Fourteen patients who had tissue remaining in storage requested a second transplantation procedure, which was performed 1‐5 years after the first graft. In 11/14 patients, this was required because of increasing FSH or reduced follicular activity, in order to improve the somewhat variable ovarian reserve and support competent oocyte recruitment. In the remaining three patients, the indication was cessation of function.

### 3.2 Duration of endocrine function

Long‐term follow‐up was conducted for up to 13 years (2006‐2019), with serial endocrine assessments until cessation of endocrine function, as defined by consistently elevated FSH levels, patients declining further surveillance or treatment, or lost to follow‐up.

The duration of ovarian activity in those whose function ceased was between 0‐65 and 0‐24 months, in groups 1 and 2, respectively. The longest functioning graft was 65 months in a patient with treated non‐Hodgkin's lymphoma, who underwent unilateral oophorectomy and cryopreservation following cyclophosphamide‐containing chemotherapy (patient 1).

### 3.3 Stimulated cycles and embryo transfers

Twenty‐eight patients underwent repeated stimulation cycles (Tables 2 and 3). The stimulation protocol evolved over time, moving away from high‐dose gonadotropins starting early in the cycle. The patients had low‐dose recombinant FSH (rFSH dose approximately 75‐112.5 IU) and GnRH antagonist, which was commenced when E2 levels were more than 250 pmol/L or follicle size reached 11 mm or more on ultrasound. Surveillance with blood tests and transvaginal (+/− transabdominal) ultrasound were conducted every 3‐4 days prior to starting medications and 2‐3 days thereafter. HCG trigger (Ovidrel^®^) was generally administered once follicular size reached 13‐18 mm.

Repeated stimulation cycles yielded a total of 193 mature (MII) oocytes. MII oocytes were retrieved from mean follicular size 14.2 mm (range 6‐20 mm) and the proportion of empty follicles was 32.2%. One hundred and two eggs fertilized with an overall 2PN fertilization rate of 55.7% (102/183; 10 MII were frozen). Thirty‐nine embryos were transferred in 12 women, resulting in 17 pregnancies, which include seven live births (five singleton and one pair of twins), two miscarriages, one ongoing pregnancy, and seven biochemical pregnancies. This equates to a clinical pregnancy rate of 25.6% (10/39 embryos transferred) and a live birth rate of 18% (7/39 embryos transferred). A number of embryos remain frozen (34), and some patients have not had an embryo transfer, as they require gestational carriers due to pelvic radiation or hysterectomy.

### 3.4 Monitoring for disease relapse

Pathology testing for malignant infiltration was performed at the time of cryopreservation and again prior to grafting. The potential risk of malignant infiltration was explained and written information was provided to patients.

In patient 10, with a history of granulosa cell tumor,^16^, ^17^ repeated laparoscopic assessments revealed no tumor recurrence and no evidence of tumor in the stored tissue. Her oncological monitoring consisted of serial pelvic ultrasounds and tumor markers, including AMH levels. She underwent ovarian tissue grafting on abdominal and pelvic sites. She became pregnant following low‐dose stimulation, IVF, and transfer of two embryos. At caesarean section, there was macroscopic evidence of tumor involving the diaphragm and a peritoneal deposit at the left pelvic brim with no evidence of tumor in the graft sites. However, the grafted tissue was removed. The histology of the resected macroscopic tumor confirmed granulosa cells as previously reported.^17^ She had no further treatment following delivery and is now in complete remission. Two patients with cervical cancer subsequently had recurrence of the cancer unrelated to the graft sites.

### 3.5 Factors predicting duration of function

The effect of tissue volume, follicle numbers, total follicular number, and age at cryopreservation on duration of ovarian function were analyzed, as shown below.

1. Tissue volume

**FIGURE 1:**
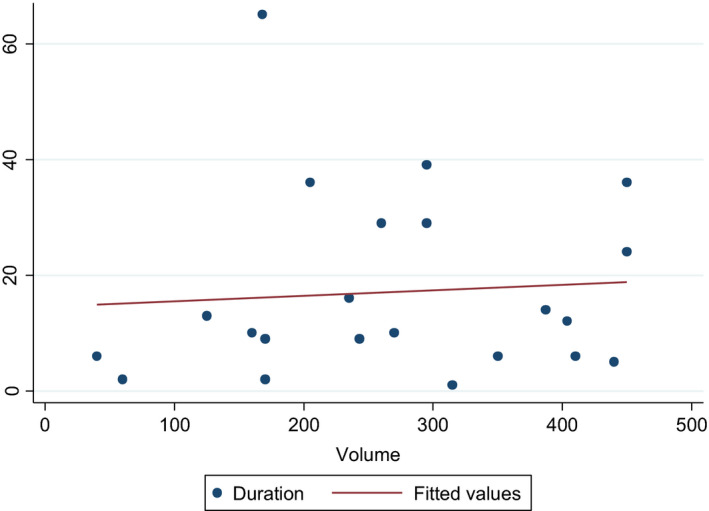
Tissue volume on duration of graft function

There was a weakly positive correlation between the duration of function and tissue volume (Pearson's *r* = .08) but this was not statistically significant (*P* = .73). This analysis excludes those whose function did not resume. Patients without tissue volume and duration of function recorded at time of harvest were also excluded. For patients with multiple grafts, the graft with the longest corresponding functional duration was used for analysis (Figure 1).

- Follicle density

**FIGURE 2:**
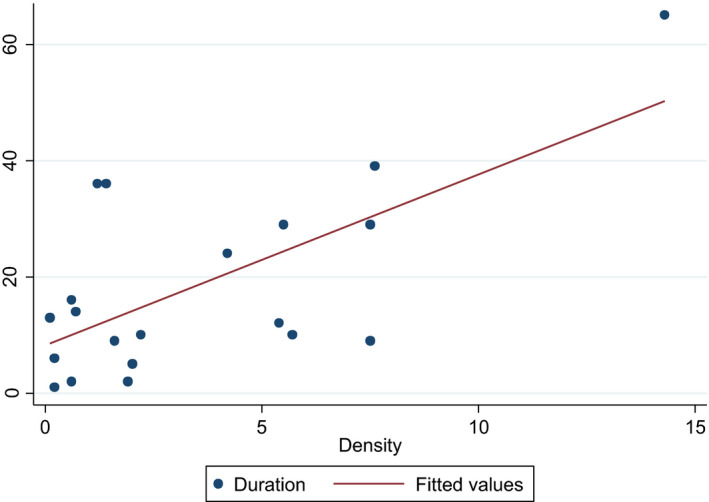
Follicular density on duration of graft function

Follicle density positively correlated with the duration of function (Pearson's *r* = .67), which was highly statistically significant (*P* = .0008). Patients without follicle density and number of follicles recorded at time of analysis were excluded. For patients with multiple grafts, the graft with the longest corresponding functional duration was used for analysis. It must be emphasized that these numbers represent an estimate only, as follicle density is based on evaluation of a very small sample of the tissue and may not be an accurate reflection of the overall density of the tissue (Figure 2).

- Total follicle number

**FIGURE 3:**
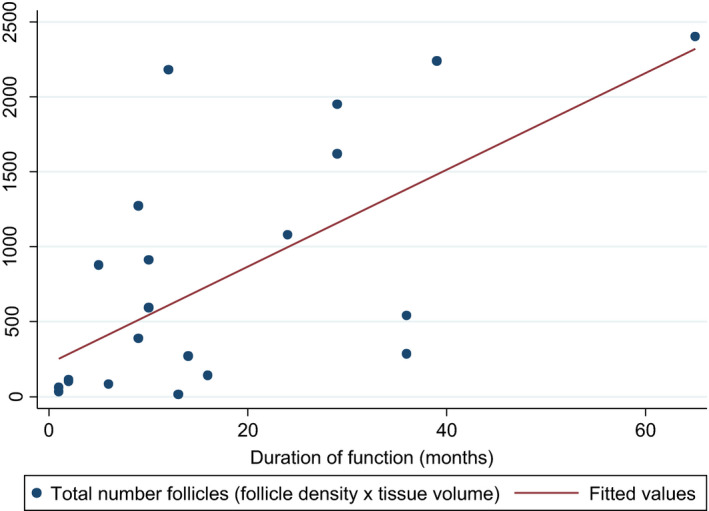
Total follicular number on duration of graft function

Total follicle number is calculated by multiplying follicle density by tissue volume. It is positively correlated with the duration of function, with Pearson's correlation of 0.63, which was highly statistically significant (*P* = .0016). Patients without follicle density and number of follicles recorded at time of analysis were excluded. For patients with multiple grafts, the graft with the longest corresponding functional duration was used for analysis (Figure 3).

- Age at cryopreservation

**FIGURE 4:**
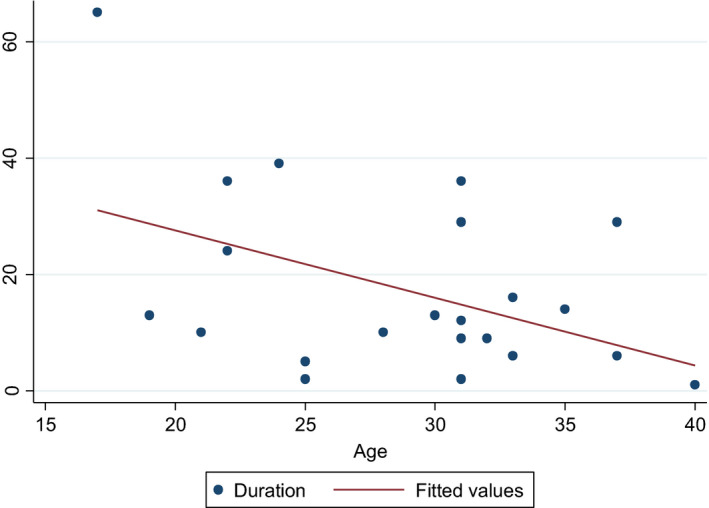
Age at cryopreservation on duration of graft function

There was a statistically significant inverse correlation between the age at harvest and duration of ovarian function (Pearson's *r* = −.46) which is statistically significant (*P* = .03). For those patients who had more than one graft procedure, the graft with the longest duration of activity was used for analysis. These data exclude those whose function did not resume and those with ongoing function (Figure 4).

### 3.6 Grafting sites

The site from which oocytes were recovered which subsequently formed embryos that resulted in a pregnancy are reported in Table 4. All were single embryo transfers except patients 9 and 10. Patient 10 had two embryos transferred, both from oocytes from the abdominal graft, which resulted in twin birth previously reported.^16^, ^17^ Patient 9 had two embryos transferred, originating from oocytes recovered from the ovarian and abdominal site, resulting in a vaginal delivery of a female infant at 34 weeks weighing 2.1 kg. Therefore, it is uncertain as to which site the pregnancy has resulted from. Her second baby was a spontaneous conception, delivered at 35‐week gestation weighing 2.6 kg. Patient 5 who had oocyte retrieval from the pelvic site delivered a healthy male infant via caesarean section at 39 weeks weighing 3.2 kg. Patient 21 who had oocyte retrieval from an abdominal site delivered a male baby via caesarean section at 39 weeks weighing 3.6 kg. Patient 13 who had oocyte retrieval from the ovary delivered a healthy female at 37 weeks weighing 2.6 kg. Patient 24 has an ongoing pregnancy, currently 36 weeks gestation. All pregnancies occurred in patients who had been aged less than 35 years at the time of tissue harvest and cryopreservation.

**TABLE 4:** Stimulated cycle outcomes

Patient no.	Oocyte collected (total MII)	Embryos transferred (total number)	Outcome	Graft site from where oocytes originate
1	23	9	1 biochemical	Pelvis
9	23	9	1 singleton live birth, 2 biochemical, 1 singleton live birth	Abdomen/Ovary (Birth) Ovary + Pelvis (2 BC, Birth)
10	6	5	1 biochemical, 1 live birth twins	Abdomen
24	20	1	1 ongoing^a^	Pelvis
33	4	2	1 biochemical	Ovary
5	10	4	1 singleton live birth	Pelvis
21	3	2	1 singleton live birth	Abdominal
2	8	3	2 biochemical	Pelvis, Abdomen
13	11	1	1 singleton live birth	Ovary
31	2	1	1 miscarriage 11 wk	Pelvic
35	57	1	1 miscarriage	Pelvic
Total	167	38	7 live births (including twins)	
			7 biochemical pregnancies	
			1 ongoing pregnancy	
			2 miscarriage	

^a^ 31 wk.

### 3.7 Effect of prior chemotherapy

Ten patients underwent chemotherapy treatment prior to ovarian tissue harvest and cryopreservation. Parameters potentially affected by chemotherapy include the follicular density, time to resumption of function and duration. The numbers are not statistically significant, and the sample size is too small to demonstrate correlation between chemotherapy and ovarian function.

## 4 DISCUSSION

Graft success, as determined by eventual pregnancy and the longevity of graft function, may be impacted by factors including age at cryopreservation, baseline ovarian reserve, techniques of tissue preparation and transplantation, history of cancer treatment, freezing‐thawing protocols, number of cortical sections grafted, transplantation techniques and graft sites, and degree of tissue ischemia after transplantation.^18^ Some studies have suggested that follicular density or ovarian tissue volume at the time of grafting could influence ovarian transplantation outcome.^3^, ^19^ Our study has shown a positive correlation between follicle density and total follicle number on duration of graft function. This could be due to the fact that patient numbers are relatively small and cancer survivors are a heterogeneous group of patients with multiple factors influencing outcome.^19^ The empty follicle rate in our study is 32.2%. Studies have shown that follicles developing in the transplanted ovarian tissue are more often without oocyte and the pregnancy rate from IVF is relatively low compared to standard IVF.^20^ An empty follicle rate as high as 23%‐35% had been reported in some studies after stimulation by gonadotropins, which could be attributed to dysfunctional folliculogenesis or damaged oocytes from freezing and thawing procedures.^20^, ^21^, ^22^ Close surveillance with ultrasound, blood tests, and earlier trigger may appear to improve the oocyte pick up rate.

The age of the patient at the time of cryopreservation is an important predictive factor of ovarian grafting success.^18^, ^23^ All clinical pregnancies in our cohort occurred in women who were aged 35 or less at the time of OTC, which is a consistent observation from other centers where most pregnant women were less than 30 years.^20^ Some studies have regarded 35 years as an upper limit for successful cryopreservation, given that the number of primordial follicles significantly decreases after this age.^3^, ^24^, ^25^ One study reported pregnancy rates of 33% following transplantation of ovarian tissue in women aged under 35 at the time of cryopreservation, in comparison to 18% in women above the age of 35.^25^ Patients of younger age at the time of gonadotoxic treatment will have higher potential for spontaneous resumption of endocrine function, given the larger baseline density of primordial follicles.^26^

One of the main challenges of ovarian transplantation is graft ischemia after the procedure.^18^ After transplantation, approximately 1/3 to 1/2 follicles suffer possible ischemic damage.^18^, ^20^ Tissue slice depth is important to regulate perfusion of cryoprotectants, oxygen and growth factors and reducing ischemic damage.^27^ Biopsy slices that are too thin can result in the absence of primordial follicles in the removed cortex.^20^ In our center, ovarian tissue is consistently prepared in thin strips of 1 mm thickness by slow freezing protocols to ensure even absorption of concentration of cryoprotectants and to prevent tissue damage.

We observed that resumption of function occurred earlier and persisted longer in the second graft compared to the first (as seen in patients 1, 4, and 23), even when there was evidence that the first graft had stopped functioning. Similarly, Rosendahl et al^29^ reported ovarian function at 2‐4 months after second grafting, which persisted for 9‐84 months. This is thought to be due to a combination of angiogenic or hormonal factors.^28^ After the initial follicular depletion in the first graft, the second graft is subject to a more stable endocrine environment and less follicular loss. The rescue mechanism is not fully understood but may be due to diffusion of paracrine substances secreted from the grafted ovarian tissue to prevent apoptosis of granulosa cells in the endogenous ovary and activate residual follicles.^30^ For this reason, like some other units, we do not use all available tissue in the first grafting procedure.^3^, ^31^, ^32^

There is no gold standard for grafting site, as it is still unclear which of the grating locations are superior.^32^, ^33^ Ovarian tissue can be transplanted onto orthotopic locations including the remaining ovary, broad ligament, or ovarian fossa, or heterotopic locations such as the abdominal wall,^34^ subcutaneous sites,^35^ rectus muscle,^36^ breast,^37^ sub‐peritoneal tissue,^15^ forearm,^38^ or renal capsule.^35^ Heterotopic transplantations can be performed via the laparoscopic, open and more recently, robotic approach.^18^, ^38^, ^39^ Heterotopic grafts allow for greater volume of ovarian tissue to be grafted in a location that is easily accessible and is considered for cases where severe pelvic adhesions preclude orthotopic transplantation. However, orthotopic grafting offers the advantage of follicular development in a more natural environment and the possibility for spontaneous conception.^40^, ^41^, ^42^ For orthotopic transplantations, whether or not the pregnancy originated from the transplanted graft or the existing ovary with remaining or recurrent ovarian activity is difficult to determine. To date, there have been more transplant procedures and births from orthotopic than heterotopic transplantation, suggesting a preference toward orthotopic grafting.^32^, ^43^ Until more robust data are available, it appears that a combination of these sites may be valuable to optimize the transplantation technique and pregnancy opportunity.

The optimal stimulation schedule for patients with grafted tissue remains unknown and presents a unique challenge, differing markedly from a ‘standard’ IVF population with respect to follicular development and numbers of oocytes retrieved. Different ART protocols have been used across various centers including spontaneous cycle, modified natural cycle, mild ovarian stimulation, and controlled ovarian stimulation.^22^ Over the years, we have moved toward stimulation with low‐dose gonadotropin use. This is based on the rationale that this group of women with low reserve and fragile ovarian function, share similarities with the low responder group of infertile women undergoing IVF.^44^ There have been some encouraging results in this group, with the use of modified natural cycles and low‐dose stimulation.^45^ This has been in line with the experience of other centers.^46^, ^47^, ^48^, ^49^ Literature reports cancer patients having a weaker response to controlled ovarian stimulation and a lower number of oocytes collected from oncology patients compared to age‐matched healthy patients,^50^ which may be due to intrinsic factors leading to reduced ovarian reserve. There is a lack of consensus of how soon ART treatment should be initiated after OTC.^22^ In our center, there is a preference toward immediate ART treatment after ovarian tissue transplantation to enable exploitation of the greater pool of follicles available given the finite lifespan of ovarian tissue grafts. A similar approach is reported by Meirow et al,^51^ which has one of the highest conception rates after ovarian tissue grafting.

Grafts from tissue prepared and frozen at other clinics showed reduced function and longevity. It is difficult to comment on why this is the case. For those who may not have local access to a unit with specialist ovarian tissue processing expertise, there is now experience worldwide with tissue transportation with a cooling device, for up to 22 hours, prior to freezing.^52^ There is an increasing number of successful pregnancies and live births from transported tissue.^9^, ^52^ This method has the potential to improve women's access to fertility preserving options, as well as standardizing outcomes between specialized and smaller centers.

A major step toward improving the overall efficacy and safety of ovarian tissue transplantation involve accessing accurate data from units performing this technique. This may be achieved by documentation of specific cycle data rather than description of results per patient as well as commitment of all centers performing grafting to comprehensive international database, where accessible information on procedures and follow‐up would be registered and regularly updated.

Transplantation of cryopreserved ovarian tissue is recognized as an established technique for fertility preservation and despite numerous publications from major centers, uncertainties still remain regarding its clinical efficacy, optimal grafting sites, factors which may predict success, as well as safety. We address these factors by reporting our experience with 55 ovarian tissue transplant procedures performed at a single center. Future direction in this field should include the optimization of transplantation reproductive outcomes, longer duration of endocrine function and improved safety profile.

## DISCLOSURES

Genia Rozen, Stephanie Sii, Franca Agresta, Debra Gook, Alex Polyakov and Catharyn Stern declare that they have no conflict of interest.

## ETHICAL APPROVAL

The protocol for the research project has been approved by a suitably constituted Ethics Committee.

## HUMAN/ANIMAL RIGHTS

The article does not contain any studies with human and animal subjects performed by the any of the authors.
